# Independent Development of Resistance to Main Classes of Anthelmintics by Gastrointestinal Nematodes of Ruminants and Horses

**DOI:** 10.3390/pathogens14090898

**Published:** 2025-09-05

**Authors:** Jacques Cabaret

**Affiliations:** Institut National de la Recherche Agronomique et Environnement (INRAE), F. Rabelais University Tours, UMR 1282, 37380 Nouzilly, France; jcabaret37@gmail.com

**Keywords:** anthelmintics, combination, ruminant, horse, nematode, resistance

## Abstract

Resistance to anthelmintics in gastrointestinal nematodes (GINs) is highly prevalent, as these parasites have been treated with anthelmintics for decades in ruminants and horses. Anthelmintics belong to different classes, each with a different mode of action. The most used are benzimidazoles and macrocyclic lactones and, to a lesser extent, levamisole and pyrantel in herbivores, as estimated from the literature. Combining these classes should be effective in controlling GIN. However, several farmers’ practices tend to promote GIN resistance. Therefore, it is unclear whether the use of anthelmintic associations is a sustainable solution for controlling resistance in natural conditions. It is not easy to establish the association of anthelmintic resistances on farms since rarely several anthelmintics and their combinations are used on a single farm. Composed probability calculations were employed when literature data indicated the presence of resistance (to benzimidazoles, levamisole, or macrocyclic lactones) in several ruminant GIN cases. The efficacy of different anthelmintics (benzimidazoles, pyrantel, or macrocyclic lactones) was evaluated in terms of the correlation between faecal nematode egg counts in horses in the available literature. No associations of anthelmintic resistance were found between the different classes of anthelmintics in either ruminants or horses. However, the association between anthelmintic resistance in GIN may appear in the long term. It is presumed that combining drugs may reduce the development of resistance and allow better control of infection on farms where resistance is already established to a low level.

## 1. Introduction

Resistance to anthelmintics of gastrointestinal nematodes has been a pervasive issue for an extended period [1,2]. Such resistance has been recorded in sheep [3,4,5,6] and goats [7,8] across numerous countries. Furthermore, evidence of resistance to anthelmintics has been documented in cattle, albeit to a lesser extent [9,10]. Extensive documentation of such cases has been recorded also in horses [11,12,13,14,15]. Most resistance studies are grounded in the faecal egg reduction test (FECRT). According to [16], a reduction of less than 90% of faecal egg counts following treatment was indicative of resistance in horses, whereas a 95% cut-off has been proposed for ruminants [17]. Resistance is typically evaluated in a control group of sheep and cattle, whereas a simple before-and-after treatment approach is recommended for horses [18]. However, it should be noted that the results of FECRT are susceptible to variation according to factors such as sampling, drug, calculation of efficacy, and level of infection [19]. In light of these variations, recent recommendations have been made to reduce this variability [17]. Another problem may obscure the evaluation of efficacy: broad-spectrum anthelmintics have a dose-limiting parasite (DLP). It means that among target parasite species listed on a product’s label, there is always one—designated as the DLP—that requires a higher dose of the active compound than the others in order to meet the efficacy standards specified on the label. Lack of efficacy may thus be due to the presence of one particular species that requires a higher dosage in a farm; it is not then a case of resistance. Bias due to DLP is found in non-strongylid nematodes of horses (*Parascaris equorum* and *Oxyuris equi*) [15].

Several facts suggest an absence of association between resistance to different anthelmintics. The targets for anthelmintic resistance mechanisms of nematodes appear to differ for benzimidazoles, imidazothiazoles, and macrocyclic lactones [20,21]. Consequently, it is anticipated that resistance to one drug should not affect resistance to another. Nevertheless, it should be noted that the precise mechanisms of resistance are not always fully elucidated, as evidenced by studies of nicotine-sensitive acetylcholine receptors [22].

Conversely, some other facts (general mechanisms of resistance and wrong use of anthelmintics on farms) suggest the presence of association between resistance to different anthelmintics. In cases where multidrug resistance mechanisms are involved [23], it is anticipated that a shared selection may occur in part and that a certain degree of association of resistance may emerge in field conditions. The apparent co-selection of benzimidazole and macrocyclic lactone in the gastrointestinal *Haemonchus contortus* [24] or *Onchocerca volvulus* in man [25] may militate furthermore for association between resistances. The management of gastrointestinal strongyles is also important for co-selecting resistant nematodes. In ovine models, Silvestre et al. [26] identified the number and timing of treatments as pivotal factors in the development of resistance. The most efficacious treatment was identified as that administered at the conclusion of winter, prior to the animals being moved again to pastures. In addition, Sallé et al. [27] identified additional risks in horses, with pastures characterised by high stocking rates and infrequent rotations being associated with resistance. Consequently, it can be hypothesised that analogous management practices may promote resistance against all anthelmintics.

A large-scale development of resistance has favoured the marketing of drug combinations. Proposals have been made for the registration of anthelmintic combinations [28]. The primary rationales for employing these combinations are twofold: firstly, to ensure effective management of nematodes in the context of single or multiple drug resistance, and secondly, to curtail the emergence of resistance to the constituent anthelmintic classes. These combinations have been commercially available for an extended period in Australia, New Zealand, South Africa, and Latin America and include dual combinations of levamisole and macrocyclic lactones, levamisole and benzimidazole, or derquantel and macrocyclic lactones. Additionally, triple combinations of benzimidazole, levamisole, and macrocyclic lactones have been utilised [29]. It has been hypothesised that these combinations may be harmful if resistances are co-selected, or, conversely, protective if there is enhanced synergy between drug metabolism [30]. Synergistic effects were identified in conjunction with derquantel (a nicotinic antagonist) and abamectin [31]. It has also been shown that the pharmacology of each drug is altered when used in combination [32], and this may modify the interest in combining drugs. The restoration of 100% efficacy of macrocyclic lactones with other classes of anthelmintics was recorded in cattle [33]. Wrigley et al. [34] observed that the combination of abamectin, levamisole, and oxfendazole exhibited high efficacy against all three species of sheep abomasal nematodes, while the combination of ivermectin, levamisole, and albendazole was not. However, it has been noticed that in cases of high resistance, the combination of anthelmintics was not effective [35]. Consequently, the outcomes of such combinations do not always align with the anticipated results. Then, there is a need to evaluate in the field how combinations are effective and relate their efficacy to the level of resistance for each drug.

It is finally difficult to assess if resistances are associated or not from similar resistance mechanisms studied in the laboratory, if these associations are due to gastrointestinal management practices in the field, or both. In fact, there are many records of multidrug resistance cases, but it is not known if it is only due to chance or if it is a true association of resistances among different anthelmintics in the field. The aim of this paper is to assess if there is a real association of anthelmintic resistances by means of statistical or probability calculations from the few selected published studies in field evaluations. Due to the limited sample size used for assessing the relation between anthelmintic associations, we compared our survey with the main traits of anthelmintic resistance to evaluate the generality of our findings.

## 2. Material and Methods

First, I will evaluate the main traits of anthelmintic resistance and their associations in a large body of literature. Then, I will present the dataset concerning ruminants, followed by the dataset concerning horses.

### 2.1. Evaluation of the Main Traits of Anthelmintic Resistance in the Literature

We concentrated on benzimidazoles, levamisole, and ivermectin that were used for decades and thus prone to high levels of anthelmintic resistance. The literature on anthelmintic resistance was screened with the Open Alex database. The analysis of the references was performed with VosViewer-1.6.20 [36]. We checked which words were associated with anthelmintic resistance, how the evolution of resistance occurred over the years, and where the most common anthelmintics were used in different countries. The percentage of papers on each anthelmintic per species of host and on resistance was obtained from screening Google Scholar.

### 2.2. The Set of Data Relating to Ruminants 

The primary data are presented in Table 1. They were chosen when at least one anthelmintic had a reduced efficacy and when there was an association of anthelmintics tested in each farm. The datasets on resistance are large for anthelmintic resistance in livestock (32,700 on Google Scholar) but our criteria of selection drastically reduced the number of available papers (16). The presence of resistance was indicated when the faecal egg reduction test result was below 90%. The 95% cut-off was utilised in two surveys conducted in New Zealand [37], and a comparison was made between these two cut-offs. The absence of associations between resistances was equally found whatever the cut-off, 90 or 95% efficacy (r_s_ = 0.99, *p* = 0.001, n = 8 [37]. The observed associations were compared to calculated ones. The latter followed the theorem of composed probabilities, which stipulates that an event A is independent of an event B if the probability of A is independent of B [38]). Therefore, the calculated associations were based on the independence of probabilities of single resistance and are outlined below. Similarly, the independence of three events (A, B, C) is determined by the condition that their joint probability is equivalent to the product of their individual probabilities. The calculated probability of association between Bz (Benzimidazoles) and Lev (Levamisole) resistance in Table 1 was determined by multiplying the frequency of Bz resistance in farms by the frequency of Lev resistance in farms. For instance, in survey one (Table 1), this is 0.56 × 0.53, i.e., 0.30 or 30%. This was then compared with an observed value of 35%. The differences between expected and observed frequencies were subjected to a statistical evaluation using a chi-square test. Their relation was also evaluated with a Spearman rho (r_s_).

### 2.3. The Set of Data Relating to Horses (Table 2)

The literature did provide FECRT for each main anthelmintic used in individual farms, but the observed associated resistances were not available in most cases. To detect the association of resistances, the FECRT of each drug in a farm or in a group of farms from the same area was correlated (Spearman r_s_). They were chosen when at least one anthelmintic had a reduced efficacy and when there was an association of anthelmintics tested in each farm. The datasets on resistance are large for anthelmintic resistance in horses (13,600 in Google Scholars) but our criteria of selection drastically reduced the numbers of available papers (11 for cyathostomins and 11 for *Parascaris*). A correlation could exist when both anthelmintics are fully efficient or when they are both poorly efficient, and that is why we selected data in which at least one anthelmintic had insufficient efficacy. The association was also compared in terms of binary data (presence or absence of resistance); benzimidazoles and pyrantel phi coefficient of correlation were calculated.

## 3. Results

### 3.1. Main Characteristics of Anthelmintic Use and Resistance

**Figure 1:**
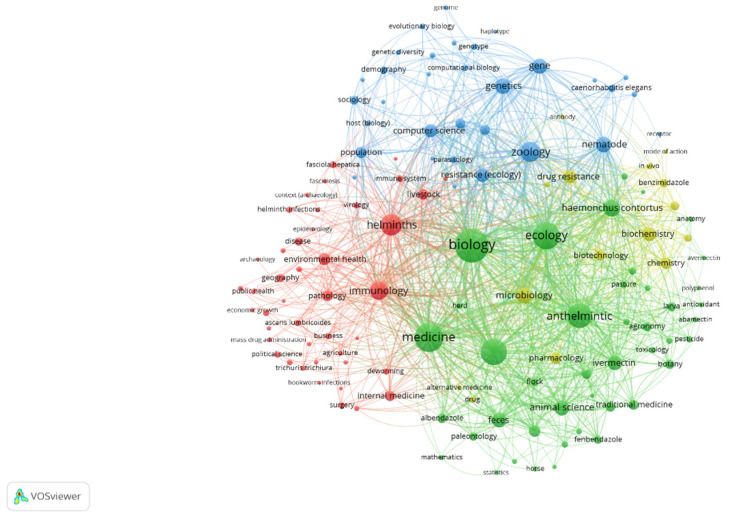
Words associated with anthelmintic and resistance with OpenAlex.

For Figure 1, the options in VosViewer were to search the queries ‘anthelmintic’ and ‘resistance’ in OpenAlex, to perform a full-text analysis, and to create a co-occurrence network visualisation. The size of the spheres and labels was related to the number of times the word occurred. The lines joining words indicate the number of associations between them. Only the main anthelmintics (benzimidazoles and ivermectin) are present in the figure. This figure shows the words associated with anthelmintic resistance: nematode (such as *Haemonchus*), benzimidazole, and mode of action. The term was also included in more general disciplines, such as zoology and ecology. Anthelmintics are associated with ivermectin, flocks, pasture, and pharmacology but not so much with anthelmintic resistance.

**Figure 2:**
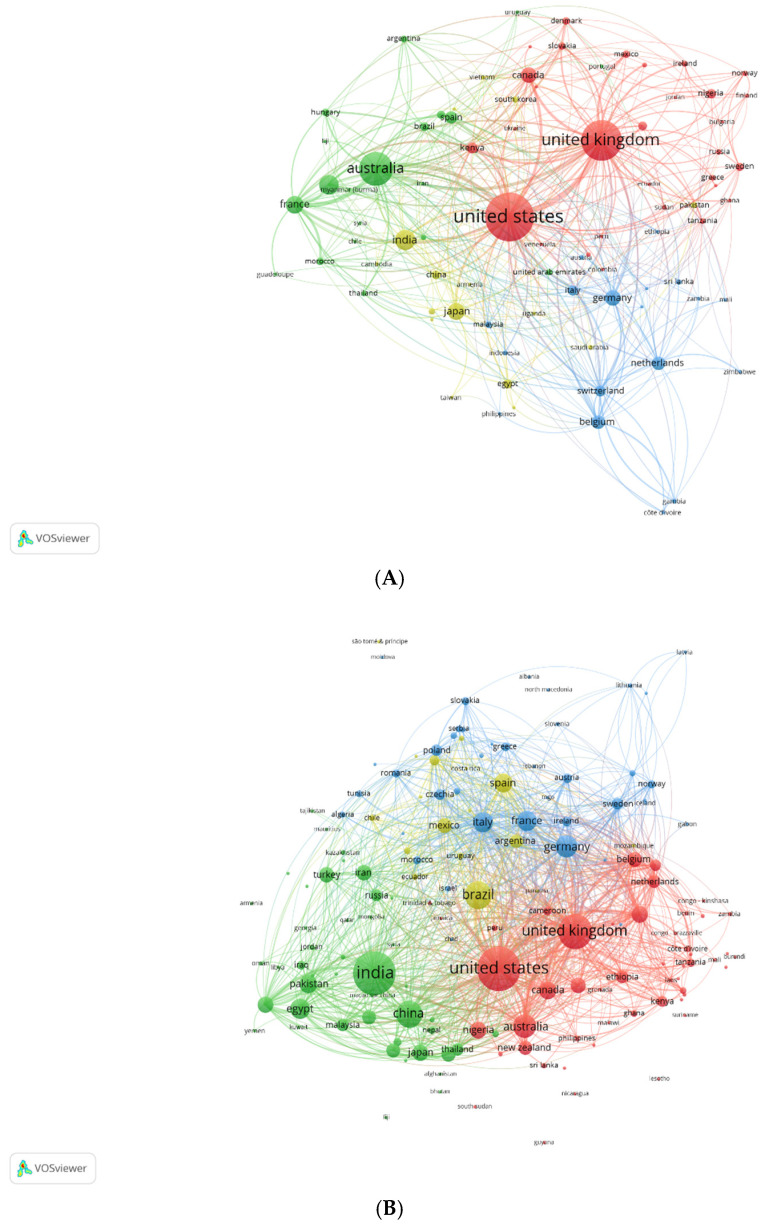
Countries publications on anthelmintics with OpenAlex: (**A**) 1960–2000 and (**B**) 2001–2025.

The prevalence of different anthelmintics in publications on anthelmintics in OpenAlex was as follows: benzimidazoles (55%), ivermectin (34%), moxidectin (7%), pyrantel (3%) and levamisole (1%). The main anthelmintics used for all species were benzimidazoles and ivermectin, both of which were considered in our selected surveys. The countries involved (2001–2025) were primarily the USA, China, India, the United Kingdom, and Germany for benzimidazoles; the USA, Brazil, and Germany for levamisole; the USA, the United Kingdom, Brazil, and India for ivermectin; and the USA, Brazil and Germany for pyrantel. Figure 2A,B (similar VosViewer conditions to Figure 1 but with the indication of countries) show how the number of publications from different countries had evolved over time: they were concentrated in a few countries in the first period (the USA, the United Kingdom, and Australia) and then became more widely distributed in recent years. It was probably indicative of the spread of anthelmintic resistance, given that it concerned publications by researchers from many more countries. The majority of the surveys we selected for further analysis were from the first few years (1960–2000) and the beginning of the 2000s for ruminants. This bias is related to the necessity of obtaining field joint evaluations of single and associated resistances on farms only present at that period.

The importance of resistance among the different hosts was evaluated based on Google Scholar references (Table 1). The total percentage of uses does not reach 100% since some drugs (e.g., moxidectin, closantel, etc.) were not investigated.

The lowest records in the literature were for levamisole in goats and cattle and for pyrantel in horses. Resistance was frequently recorded for all four drugs: 56–100%. Combination therapy in herbivores was cited in 3980 Google Scholar results, including 799 reviews. However, in research papers, the combination was often only cited as a possibility and not investigated. This means that I had to select manually the references of interest.

### 3.2. Anthelmintic Associations in Ruminants (Table 2)

**Figure 3:**
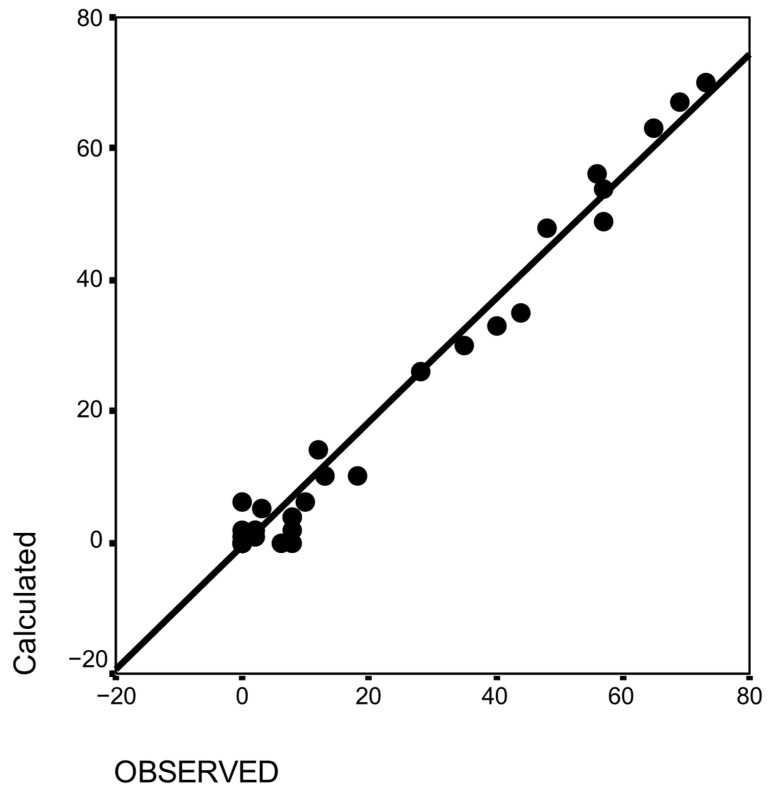
Relationship between observed and calculated associations of anthelmintic resistances in sheep.

The observed and calculated frequencies of all associated resistance were highly related (see Figure 3 for all drug associations in all surveys). The chi-square for each type of association was not significant (*p* = 0.89 to 0.99). Thus, we calculated for all the associations a general Spearman r_s_; it was 0.90 (*p* < 0.0001; n = 55): the observed and calculated associations were highly similar.

### 3.3. Anthelmintic Associations in Horses

#### 3.3.1. Strongyles

Strongyles were mostly cyathostomins. A calculation similar to that of ruminant on observed and calculated association of resistance was performed on the data of Table 3. There was no significant difference even for the Denmark survey [48].

**Figure 4:**
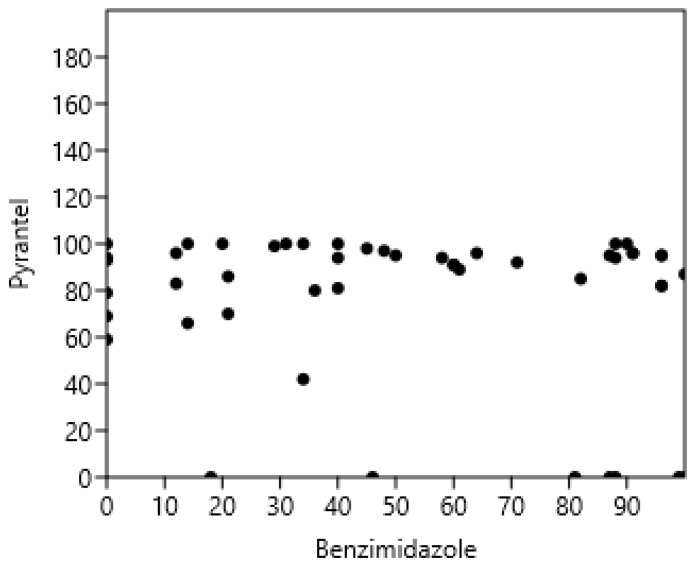
Absence of correlation between pyrantel and benzimidazoles faecal strongyle egg reduction test in horses.

The results of the FECRT for each farm are presented in Table 4. The FECRT results for ivermectin were high on all farms and could therefore not be correlated with the other resistances. There was no significant relationship between pyrantel and benzimidazole FECRT (r_s_ = −0.06; *p* > 0.05; n = 55): see Figure 4. Analysing the data in Table 4 in terms of resistance or not, based on FECRT < 90%, showed no relationship between resistance to Bz and Pyr (Phi coefficient 0.27, *p* > 0.05).

#### 3.3.2. Parascaris

The data on *Parascaris* are presented in Table 5.

**Figure 5:**
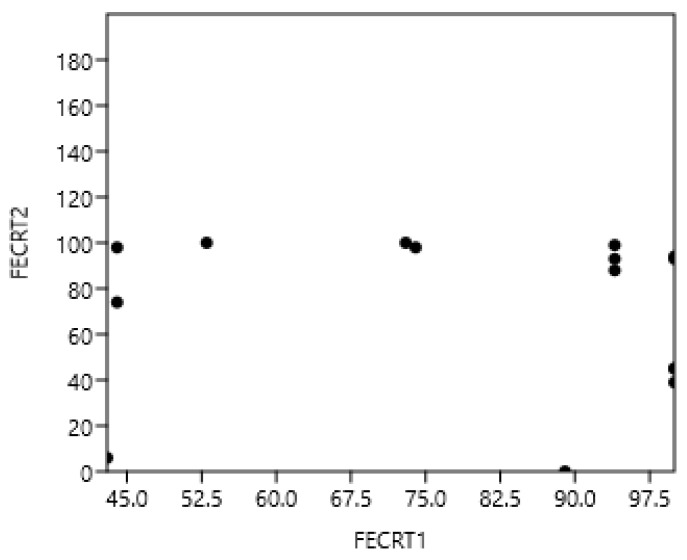
Absence of correlation between benzimidazoles, pyrantel (1), and ivermectin (2) in the faecal *Parascaris* egg reduction test (FECRT) in horses.

The Spearman correlations between FECRT of the three drugs (r_s_ = −0.03, n = 22, *p* = 0.92) were not significant (Figure 5). The correlations between benzimidazoles and pyrantel (r_s_ = −0.12), benzimidazoles and ivermectin (r_s_ = −0.22), and pyrantel and ivermectin (r_s_ = 0.54) were not significant.

## 4. Discussion

First, I will discuss the necessity of combining anthelmintics due to the increase in resistance. As few new anthelmintics appear on the market, molecules released many years ago, such as levamisole and pyrantel, may be included in these combinations. Secondly, I will present ways to evaluate the efficacy of these combinations in field conditions. Two methods will be employed and evaluated: a combined probability method and a regression method. Thirdly, I will present the absence of a relationship between resistance to the different tested anthelmintics, based on our data. Fourthly, the pros and cons of combining anthelmintics will be described.

### 4.1. The Necessity of Combining Anthelmintics

Since anthelmintic resistance is a growing concern worldwide (Figure 2A,B, ref. [1,68]) and new anthelmintics available are scarce; a combination of existing drugs has been proposed. The examination of the literature gives a false idea of the importance of the works on combinations of anthelmintics. Many are presented in review articles and based on the difference in mode of action of each anthelmintic class. The most studied are benzimidazoles and macrocyclic lactones (such as ivermectin and moxidectin), which are used in all species of hosts (Table 1). Levamisole (mostly in sheep) and pyrantel (only in horses) did represent a very small part (a few percent of the literature) (see Table 1) and were more cited in older works. They regained interest, probably due to nowadays anthelmintic associations used to cope against GIN resistance. I selected benzimidazoles, ivermectin, and levamisole in ruminants and benzimidazole, ivermectin, and pyrantel in horses because I could obtain data with measured individual and associated drug resistances. I chose to evaluate drugs that have been used for decades because they give an indication of the potential for joint resistance to build up.

### 4.2. How to Evaluate Efficacy of Combinations?

Assessing the efficacy of combinations of different classes of anthelmintics is not a straightforward task. When two drugs are combined, they may behave like a third, introducing a range of uncertainties. The efficacy of oxfendazole and pyrantel was relatively low, whereas abamectin and morantel showed high efficacy in horses [67], depending on the history of resistance to these drugs. In theory, the resulting Combination Index (CI), based on the Chou-Talalay theorem, provides a quantitative definition of the additive effect (CI = 1), synergism (CI < 1), or antagonism (CI > 1) of drug combinations [69,70]. The proposed method for assessing the CI is experimental and requires efficacy measurements at various doses of each drug and their combinations, expressed as percentages [69]. This approach was applied in an animal model to evaluate the most effective drug combinations for treating *Trichuris* in humans [71], but it is not feasible in routine practice.

In many current trials, each drug’s efficacy is assessed separately at the recommended dose, which means no information is obtained on the efficacy of combinations or resistance patterns. However, when drugs are used concurrently against gastrointestinal nematodes (GIN) and prevalence of resistance is established in a large number of farms, I demonstrated that the efficacy of the combination can be estimated using probability calculations and compared to the actual results. This was true for many of the ruminant studies I examined. In contrast, combination efficacy studies in horses were limited; only three were comparable to those in ruminants (Table 3). Therefore, alternative strategies were employed for horses, such as correlating the faecal egg count reduction test (FECRT) results for different drugs in a farm. The correlation of FECRT should be used with caution since if all drugs are efficient or none, the correlation is high; I avoided correlation due to the absence of resistance by selecting farms with at least one resistant anthelmintic. One issue with my evaluation of associations is that the FECRTs were not evaluated using the same methods, which could have introduced bias into the efficacy results. This was probably more significant for the correlation method than the probability method: the former considered the exact value of the FECRT, whereas the latter assessed resistance as efficacy below 90%. The probability method can also be understood in the context of repeated experiences, where variability is divided by the square root of the number of experiences (here, four for ruminants). The ruminant results are more reliable than the cyathostomin horse results (larger number of farms and better evaluation of associations). The data concerning *Parascaris* might be the least reliable since this species is a dose-limiting parasite [15]. This selection of surveys was performed on an automatic selection (Google Scholar and AlexOpen) and then screened manually to find the information I needed.

### 4.3. The Independence of Resistance to Major Anthelmintics

In all cases (Figure 3, Figure 4 and Figure 5), resistance to different classes of anthelmintics and for different nematodes (GIN of ruminants, Cyathostomins, and Parascaris in horses) was not associated in the field as it might be expected based on their molecular targets in nematodes [21]. Although it is based on a limited number of references, it encompasses the results of more than 1200 ruminant farms and 120 horse farms, making it representative of the actual situation regarding resistance. However, cases in New Zealand cattle suggest that the resistance to oxfendazole, levamisole, and macrocyclic lactones could emerge simultaneously under the strong selective pressure of these drugs [72]. There is also a record of associated resistance between eprinomectin and doramectin in the USA [73]. In the long term, it is possible that associations of resistance to multiple classes of anthelmintics will emerge: (i) if no new anthelmintic is available, and (ii) an intensive use of the available anthelmintics is promoted as the only manner of controlling GIN, then (iii) resistance will then appear progressively in all anthelmintics after being sequentially used.

The appearance of resistance is due to human (choice of anthelmintics, frequency of treatments, etc.), genetic (selection of mutations in worms), and environmental factors (choice of pasture to preserve refugia). The farmers’ practices concerning anthelmintic use and animal management frequently do not follow the requirements based on research [74]. In theory, this should have led to the appearance of drug resistance, but this was not evident in our data. Most factors influencing the emergence of resistance—such as treatment frequency [73] and timing in relation to worms in refugia [75]—are based on benzimidazole resistance and may differ for other drug classes. It is also difficult to pinpoint the most probable reason for the appearance of resistance. In dairy ewes in south-east France, for example, transhumance was identified as the main risk factor for the emergence of resistance to the macrocyclic lactone eprinomectin, likely due to the spread of resistant GIN between flocks [76]. Hence, the various husbandry management scenarios may explain why anthelmintic resistances are not necessarily related.

### 4.4. The Pros and Cons of Combining Anthelmintics

The principle that combinations of chemotherapeutic agents can benefit infected hosts by maintaining drug efficacy in the presence of resistance has been repeatedly demonstrated for various pathogens, drawing on insights from insecticide and pesticide use [28]. In ruminants—particularly sheep—combinations of two or more anthelmintics are primarily used to manage resistance [77]. They may delay the emergence and spread of resistance [28] and help maintain control of GIN populations despite existing resistance [29]. Modelling studies have also indicated that combinations provide significant advantages for both drugs when used together, especially if they initially have high efficacy [78]. Using anthelmintics in combination has been shown to be superior to using them individually, whether in sequence or rotation, even when some cross-resistance exists between the two classes [79]. However, concerns remain regarding the use of combinations, particularly the potential risk of selecting for multi-class resistance if (i) refugia (GIN not submitted to anthelmintics) are insufficient and (ii) the initial frequency of resistance alleles is too high on certain farms to justify introducing combination treatments [79,80]. When resistance is high for several anthelmintics, farmers should not rely on the sole anthelmintic treatment and turn to other control methods (among others: use of predatory hyphomycetes or plants extracts [81].

## 5. Conclusions

It is difficult to establish associations—whether positive or negative—between anthelmintic resistances across different drug classes in field conditions. Such associations can be explored by calculating the probability of cross-resistance (when tests on individual anthelmintics and their combinations are available in different farms) or by cautiously evaluating the correlation between drug efficacy (when no combination is tested), as determined by faecal egg count reduction tests. No clear association of resistance has been demonstrated under farming conditions, either in ruminants or in horses. Therefore, the use of anthelmintic combinations may be of interest as a strategy to possibly slow the development of resistance and to maintain effective control of GIN infections when resistance has not yet reached a high level. Research should focus on designing the most effective anthelmintic combinations in relation to pre-existing resistance status and on understanding their potential pharmacological implications on efficacy.

## Figures and Tables

**Table 1:** Anthelmintic use and resistance based on references in Google Scholar.

Anthelmintic	Hosts	% of Papers on Use of Anthelmintic (Number of Papers with One Anthelmintic in a Host Species/Number of Papers on All Anthelmintics)	% of Resistance (Number of Papers on Use/Number of Papers on Resistance)
Benzimidazoles	Sheep	10.5	76
	Goat	8.4	56
	Cattle	9.5	74
	Horse	6.0	74
Levamisole	Sheep	10	70
	Goat	2.0	100
	Cattle	1.0	100
Ivermectin	Sheep	15.0	73
	Goat	10.4	71
	Cattle	9.5	63
	Horse	9.3	64
Pyrantel	Horse	2.4	76

**Table 2:** Gastrointestinal resistance to the three main groups of anthelmintics in 1265 ruminant farms (Bz: benzimidazoles, Lev: levamisole/tetramisole imidazothiazoles, Ml: ivermectin, a macrocyclic lactone).

Survey			Resistance Based on FECRT < 90%						
			% of Farms with Resistance to			% of Farms with Association Between Resistances			
Host	No Farms	Country (Reference)	Bz	Lev	Ml	BzLev	BzMl	LevMl	BzLevMl
Goats	34	Brazil [39]	56	53	nd *	35	nd	nd	nd
	18	France [40]	67	0	nd	6	nd	nd	nd
Sheep and goats	25	Brazil [41]	92	68	52	65	48	44	40
Sheep	10	Algeria [42]	50	nd	8	nd	8	nd	nd
	10	Morocco [43]	50	10	1	3	1	0	0
	336	Australia [4]	86	65	0	56	0	0	0
	118	Australia [4]	93	72	0	69	0	0	0
	56	Australia [4]	89	29	0	28	0	0	0
	315	Australia [4]	81	67	0	57	0	0	0
	56	Australia [4]	88	80	0	73	0	0	0
	80	New-Zealand [37]	41	24	25	18	13	10	8
	85	Spain [44]	13	9	16	1	0	2	0
	23	France [45]	61	17	0	13	0	0	0
Cattle	13	Argentina [46]	8	0	54	0	8	0	0
	25	Brazil [47]	20	8	70	0	12	0	8
	61	New-Zealand [37]	76	8	92	8	74	8	5

* nd: not done.

**Table 3:** Strongyle resistance to two anthelmintics (Bz: benzimidazole, Pyr: pyrantel) in 66 horse farms.

Survey Location (No of Farms)	Reference	% of Resistant Farms Based on FECRT < 90%f			
		Bz	Pyr	BzPyr Observed	BzPyr Calculated
Denmark (24)	[48]	82	42	16	34
Norway (17)	[49]	94	12	12	11
Sweden (25)	[50]	100	28	28	28

**Table 4:** Efficacy on gastrointestinal nematodes of the three main groups of anthelmintics in 56 individual horse farms (Bz: benzimidazoles, Pyr: pyrantel, Ml: ivermectin, a macrocyclic lactone).

Survey		FECRT (%)		
Country	Reference	Benzimidazole	Pyrantel	Ivermectin
Brazil	[51]	14	66	99
		61	89	96
		0	94	100
		0	59	89
		0	93	99
		0	79	96
		12	96	91
		29	99	98
		0	69	100
		40	94	100
		48	97	100
Denmark	[48]	46	0	nd *
		100	0	nd
		87	0	nd
		18	0	nd
		100	87	nd
		99	0	nd
		81	0	nd
		88	0	nd
		71	92	nd
		34	100	nd
France	[27]	40	81	99
		45	98	94
		50	95	100
	[52]	60	nd	96
		45	nd	100
	[53]	87	95	100
		82	85	100
		21	86	100
		20	100	100
		58	94	100
		88	100	100
		88	94	100
		0	100	100
		14	100	100
		43	100	100
		40	100	100
		64	96	100
		90	100	100
		31	100	100
Italy	[54]	60	91	100
		96	95	100
		96	82	100
		91	96	100
Mexico	[55]	89	nd	98
		93	nd	100
Morocco	[56]	16	95	nd
Norway	[49]	60	91	100
		96	95	100
		96	82	100
		91	96	100
Ukraine	[57]	69	nd	100
		99	nd	100
		97	nd	100
USA	[58]	36	80	99
		21	70	99
		34	42	99

* nd: not done.

**Table 5:** Efficacy on *Parascaris* of three main groups of anthelmintics in 60 horse farms.

Survey		FECRT (%)		
Country (No of Farms)	Reference	Benzimidazole	Pyrantel	Ivermectin
Argentina (1)	[59]	100	nd *	39
Australia (1)	[60]	44	74	98
Brazil (1)	[61]	100	94	93
Brazil (9)	[62]	89	0	nd
		94	99	nd
		53	100	nd
		73	100	nd
Estonia (4)	[63]	nd	94	88
France (18)	[61]	100	100	45
Finland (1)	[64]	nd	52	43
Sweden (9)	[50]	100	90	nd
USA (7)	[65]	84	0	0
USA (8)	[66]	80	2	nd
USA (1)	[67]	nd	97	47

* nd: not done.

## Data Availability

Data are available within this paper.
